# Preparation of the Branch Bark Ethanol Extract in Mulberry *Morus alba*, Its Antioxidation, and Antihyperglycemic Activity *In Vivo*

**DOI:** 10.1155/2014/569652

**Published:** 2014-01-22

**Authors:** Shu Wang, Meng Fang, Yong-Lei Ma, Yu-Qing Zhang

**Affiliations:** Silk Biotechnology Key Laboratory of Suzhou City, Medical College of Soochow University, Suzhou 215123, China

## Abstract

The biological activities of the branch bark ethanol extract (BBEE) in the mulberry *Morus alba* L. were investigated. The determination of active component showed that the flavonoids, phenols, and saccharides are the major components of the ethanol extract. The BBEE had a good scavenging activity of the 1,1-diphenyl-2-picrylhydrazyl (DPPH) radical with around 100 **μ**g/mL of IC_50_ value. *In vitro* assay revealed that the BBEE strongly inhibited both **α**-glucosidase and sucrase activities whose IC_50_ values were 8.0 and 0.24 **μ**g/mL, respectively. The kinetic analysis showed that the BBEE as a kind of **α**-glucosidase inhibitor characterized a competitive inhibition activity. Furthermore, the carbohydrate tolerance of the normal mice was obviously enhanced at 0.5 h (*P* < 0.05) and 1.0 h (*P* < 0.05) after the BBEE intragastric administration as compared to negative control. At 0.5, 1.0, 1.5, and 2.0 h after the intragastric administration with starch, the postprandial hyperglycemia of the type 2 diabetic mice can be significantly decreased (*P* < 0.01) by supplying various concentrations of the BBEE (10–40 mg/kg body weight). Therefore, the BBEE could effectively inhibit the postprandial hyperglycemia as a novel **α**-glucosidase activity inhibitor for the diabetic therapy.

## 1. Introduction

Diabetes mellitus is a metabolic disorder characterized by a congenital (type 1 insulin-dependent diabetes mellitus/IDDM) or acquired (type 2 noninsulin-dependent diabetes mellitus/NIDDM) inability to transport glucose from the bloodstream into cells. The type 1 is usually diagnosed in childhood, and the body makes little or no insulin. The type 2 diabetes is an insulin resistance condition that occurs in adulthood, and it afflicts approximately 90% of all diabetics. The most beneficial therapy for the type 2 is said to be one that achieves optimal blood glucose control after a meal [1]. Now the pharmacological agents with the greatest effect on postprandial hyperglycemia included insulin, lispro, amylin analogues, and *α*-glucosidase inhibitors such as Acarbose, Voglibose, Miglitol, and Emiglitate [2] in which Acarbose had been extensively used in clinical practice. But the oral hypoglycemic agents currently used in clinical practice have serious side effects [3]. Management of hyperglycemia or hyperlipidemia with low side effects is still a challenge to the current medical system. Hence, there is a need to search for newer antidiabetic agents that retain therapeutic efficacy and are devoid of side effects. It is necessary to identify and have a large pool of the natural resources. There were many articles related to antidiabetic compounds from plants [4, 5]. It has been reported that about 800 plant species mostly possess strong antioxidant [6], antidiabetic [7] and multiple therapeutic properties [8]. At present, the natural extracts as *α*-glucosidase inhibitors have been paid more attention due to the few side effects or no harm to human bodies.

Mulberry trees (*Morus alba* L.) were extensively grown in sericultural countries (e.g., China, India, Korea, Thailand, and Brazil) for their leaves as food for silkworms *Bombyx mori*. More than ten million tons of the mulberry branches harvested only in China were used for firewood or agro-wastes every year. These enormous biological resources have been not utilized and regenerated in the world. In fact, the mulberry leaves and fruits were early established to be a medical material and a food, as well as its roots used for the medicine material and its branches were a healthy food material that were established in Pharmacopoeia of the People's Republic of China (2000 edition). In Korea and Japan, mulberry leaves were largely consumed by diabetic patients as an antihyperglycemic nutraceuticals, because they contained the 1-deoxynojirimycin (DNJ) [9] and the flavonoids which were known as two of the most potent *α*-glucosidase inhibitors [10]. Even the freeze-drying powder of silkworm fed on mulberry leaves was also the blood glucose-lowering agent [11–13]. In addition, the bark of mulberry root had been always used as diuretic, cough medicine, expectorant, and antifebrile and for the treatment of bronchitis, asthma, vomiting, beriberi, and dropsy in traditional Chinese medicine. Recent researches showed that it also had anti-HIV, antioxidative, antihypotensive, and cytotoxic activities [14]. If these branches can be used as a stock for processing anti-hypoglycemic agents, it will make great contributions to the recycling of mulberry bioresource for human being and blood glucose control of diabetes. Up to now, there are few reports on the extract of mulberry branch or its barks as a novel *α*-glycosidase activity inhibitor.

In the present work, we described the preparation, properties, and biological activities of the branch bark ethanol extracts (BBEEs) in the mulberry. In particular, the antihyperglycemic activity of the BBEE was investigated *in vitro* and *in vivo* as a novel *α*-glucosidase inhibitor.

## 2. Materials and Methods

### 2.1. Materials

The *α*-glucosidase (EC.3.2.1.20) from the rat intestinal acetone powder, tyrosinase, and streptozocin (STZ) were purchased from Sigma-Aldrich Fine Chemicals (CMO, USA). 1,1-Diphenyl-2-picrylhydrazyl (DPPH) was obtained from Calbiochem. Rutin trihydrate, maltose, and sucrose were purchased from Shanghai chemical reagent Co. Ltd. Glucose reagent kit was purchased from Shanghai Shensuo Reagent Co., Ltd. (China). All of other chemicals and solvents used were of analytical grade.

Total 50 of the male Kunming ablactation mice (18–22 g BW) with three weeks old were provided from the Experimental Animal Center of Soochow University. The study was approved by the Institute Review Board (IRB) of Soochow University, and the number of the IRB approval of the research is IRB no. 11-01-0020.

### 2.2. Preparation of Sample

The mulberry trees (*Morus *L) grow in the mulberry garden of Soochow University, Suzhou, China. Nong-Sang no. 8 is a cross-breeding cultivated mulberry from *Morus alba* L and its branches were harvested in November 2010. The barks peeled from mulberry branches were dried in an oven at 100°C for 2 h and then were pulverized. The bark powder was extracted repeatedly with 30% ethanol solution for 3 times, the ethanol in extractive solution was recovered by a spinning concentrator, and the concentrated solution was again extracted with a mixture of chloroform and n-butanol (4 : 1, V/V). And then the extractive solution was treated in active carbon to remove some pigments. Finally the extracted solution was made into the powder with a spraying dryer (Shanghai Trustech Co. Ltd.) and the resulting powder of the branch bark ethanol extract (BBEE) was stored at −20°C for the following experiments.

### 2.3. Determination of Active Components in the Extract

Total contents of the flavonoids were determined according to the method described previously by Jia et al. [15]. Briefly, an aliquot (50 *μ*L) of the BBEE or standard solution was taken to mix with a 10 *μ*L of 10% NaNO_2_ solution. After 6 min, a 10 *μ*L of 20% AlCl_3_ in water was added. Waiting for 5 min, a 50 *μ*L of 2 M NaOH solution was added, and then the total volume was fixed to 320 *μ*L with 60% ethanol (V/V). After being mixed thoroughly and stood for 15 min, the absorbance of the mixed solution against blank was determined at 510 nm. Rutin trihydrate was utilized for constructing the standard curve (2–10 *μ*g/mL; *r* ^2^ = 0.9989; *y* is the absorbance; *x* is the solution concentration). An UV spectrophotometric method was used for the determination of total phenols (TP) with gallic acid as equivalents [16]. Gallic acid was utilized for constructing the standard curve *y* = 0.014*x* + 6.316*E* ^−4^ (0~100 *μ*g/mL; *r* ^2^ = 0.999). Total saccharides (TS) were measured in spectrophotometry using glucose as a standard by the method [17] described previously with a slight modification [18]. Glucose was utilized for making the standard curve *y* = 0.011*x* − 0.003 (0~60 *μ*g/mL, *r* ^2^ = 0.994).

### 2.4. Measurement of Scavenging Activity of DPPH Radical

The assay for scavenging activity of the stable free radical (DPPH) was carried out as reported earlier [19] with some modifications. The stock solution of the BBEE was diluted to the final concentrations of 2.00, 1.00, 0.67, 0.50, 0.33, 0.20, and 0.10 mg/mL, respectively. In brief, a 80 *μ*L of the various concentrations of the test sample that dissolved in the absolute ethyl alcohol or in a 80 *μ*L of ethanol (as the control) and a 80 *μ*L of 0.4 mM DPPH dissolved in absolute ethyl alcohol were added into a 96-well micro-plate and then the reaction mixture was shaken well and kept at room temperature for 30 min. The absorbance was read at 550 nm. The percentage of the DPPH-scavenging activity was expressed as [(absorbance of control sample − absorbance of test sample)/absorbance of control sample] × 100 (%).

### 2.5. Determination of Tyrosinase Inhibition

The tyrosinase-inhibition activity was determined by using L-tyrosine as substrate. A 10 *μ*L of mushroom tyrosinase (200 units/mL) solution and a 10 *μ*L of the BBEE were added into a 96-well microplate. The mixture was preincubated at 30°C for 10 min and added 280 *μ*L of 0.5 mM L-tyrosine; then, the assay mixture was incubated at 30°C for 15 min. The control sample was prepared by adding of the phosphate buffer (pH 6.8, 50 mM) instead of the extract. The absorbance of the reaction mixture was measured at 492 nm in a 96-well microplate. The data were expressed as a percentage of inhibition of tyrosinase activity.

### 2.6. Determination of *α*-Glucosidase and Sucrase Inhibitory Activity

The *α*-glucosidase or the sucrase inhibitory activity was assayed by the method [20] described previously with a slight modification. 100 *μ*L of the BBEE solution, 700 *μ*L of phosphate buffer (pH 6.0, 100 mM), and 100 *μ*L of 50 mM maltose or sucrose solution were added into a test tube. After incubation of the mixture at 37°C for 5 min, added a 100 *μ*L of *α*-glucosidase or sucrase from rat intestine solution (a stock solution of 100 *μ*g/mL in 0.9% physiological saline that was diluted 4-times with deionized water) into the reaction mixture which was incubated for 60 min at 37°C; then, the reaction was stopped by adding 1000 *μ*L of 1.0 M Na_2_CO_3_. The control sample was prepared by adding phosphate buffer (100 mM, pH 6.0) instead of the extract. The *α*-glucosidase or sucrase inhibitory activity was estimated by the difference of liberated glucose with or without the BBEE. Determination of glucose was performed with glucose reagent kit. The percentage of *α*-glucosidase or sucrase inhibition was calculated as follows:

(1) $$\begin{array}{c} \text{Inhibitory}\;\text{Ratio}\;\left(\%\right)=\frac{\left[\left(A_{C}-A_{\text{CB}}\right)-\left(A_{S}-A_{\text{SB}}\right)\right]}{\left(A_{C}-A_{\text{CB}}\right)}\times 100, \end{array}$$

where *A*_*C*_, *A*_*S*_, *A*_*B*_, *A*_CB_, and *A*_SB_ represent the absorbance of the control, sample, blank, control blank, and sample blank, respectively. The IC_50_ value under the assay condition is defined as the concentration of *α*-glucosidase or sucrase inhibitor required inhibiting 50% of the *α*-glucosidase or sucrase activity. The inhibition activity of each sample was determined repeatedly for 3 times. The resulting data were calculated with the same method as mentioned above.

### 2.7. Kinetics of *α*-Glucosidase Inhibitor

The Lineweaver-Burk plots for *α*-glucosidase inhibition of the BBEE were performed by the reported assay [21]. The substrate was maltose, and the final concentration of the maltose was changed (0.5, 1.0, 2.5, and 5.0 mM) while keeping the inhibitor concentration constant (1.95 or 1.56 *μ*g/mL). All samples were measured repeatedly three times and the values were expressed as the mean ± standard deviation (±SD, *n* = 3). The linear equation and the correlation efficient of the BBEE as a *α*-glucosidase inhibitor were estimated by using Origin 7.5 software.

### 2.8. Carbohydrate Tolerance Test

For the oral carbohydrate tolerance test, the male Kunming mice weighing 19~22 g were divided at random into five groups of 10 each. The animals were deprived of food overnight before being intragastrically administered. The animals are intragastrically administered starch 10 g/kg, with or without the BBEE. The first group was the negative control (received 0.9% physiological saline, group A) and the second was the positive control treated with the Acarbose at a dose of 10 mg/kg (group B). The mice were treated with the BBEE at the doses of 5, 10, and 30 mg/kg (group C, group D, and group E), respectively. The blood was sampled from the tail vein at 0, 30, 60, 90, and 120 min after carbohydrate administration to measure the blood glucose levels by a portable OneTouch glucometer (LifeScan OneTouch SideStep Meter, USA; range 1.1~33.3 mM).

### 2.9. Construction of STZ-Diabetic Model

The male Kunming mice weighing 19~22 g were obtained from the Center of Laboratory Animal of Soochow University (Suzhou 215123, China). After an overnight fast, the mice were tail intravenous administered a single dose injection of 100 mg/kg STZ dissolved in a freshly prepared 0.1 mM citrate buffer (pH 4.5) and injected within a few minutes to avoid degradation. Nondiabetic control mice were injected with citrate buffer alone. After being injected, all of the mice continued a standard diet for one week. The development of the hyperglycemia in mice was confirmed by fasting blood glucose estimation one week after STZ injection. The blood glucose was estimated for overnight fasted mice during the daily visit (8:00~9:00) with a portable OneTouch glucometer using a drop of blood from the tail vein. The animals with fasting blood glucose level of above 7.8 mM were considered as diabetic and only uniformly diabetic mice were used in the following experiment.

### 2.10. *In Vivo* Test

The STZ-diabetic mice were divided at random into five groups of 10 each. Before being intragastrically administered, the mice were deprived of the food overnight. The first group was the control (received citrate buffer alone, group A) and the second, the third, the fourth, and the fifth groups were the STZ-diabetic mice treated with the BBEE at doses of 10, 20, 30, and 40 mg/kg (group B, group C, group D, and group E). The blood was sampled from the tail vein at 0, 30, 60, 90, and 120 min after intragastrical administrating carbohydrate and the extracts or citrate buffer to measure the blood glucose levels by a portable OneTouch glucometer.

### 2.11. Statistical Analysis

The statistical significance of the data obtained was evaluated by Student's *t*-test using unpaired analyses. The data are presented as the mean ± standard error for the indicated number of independently performed experiments.

## 3. Results 

### 3.1. Active Components of the Extracts

In this study, the rutin, gallic acid, and glucose were used as the reference standards to determine the total flavonoids (TF), phenols (TP), and saccharides (TS) contents in the sample extracts, respectively. Their standard curves were drawn with the qualities (*lg*) of the rutin, gallic acid, and glucose as the abscissa and the absorbance values at 510 nm, 760 nm, and 490 nm as the ordinate, respectively. As shown in Table 1, TF, TP, and TS contents were calculated by using these linear regression equations. The results showed that TF, TP, and TS contents in the extract were 262.16, 118.70, and 75.88 mg/g, respectively. Total flavonoids level was the highest of the three active components in ethanol extracts. Total contents of the flavonoids and phenols could reached about 40% of the ethanol extracts. Therefore, the flavonoids, phenols, saccharides, and flavonoids glycosides are the major components of the ethanol extract.

### 3.2. The Free-Radical Scavenging Activity

The antioxidants are important for diabetes, as the low levels of the plasma antioxidants are implicated as a risk factor for development of the disease. Therefore, the antioxidant action of the medicinal plants associated with the antidiabetic properties may be an important therapeutic property [6].

**Figure 1:**
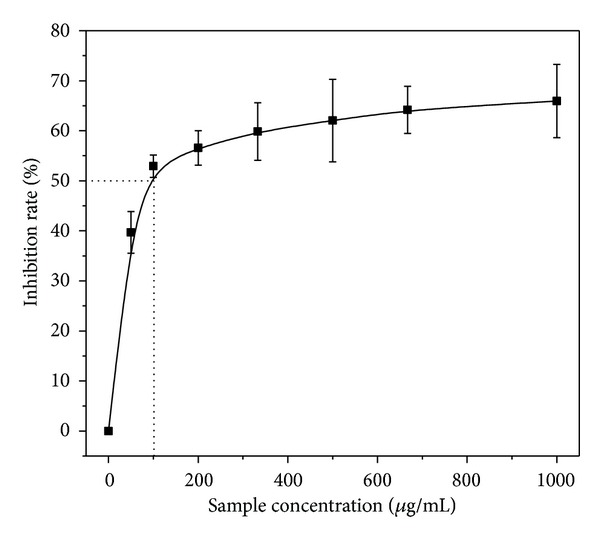
DPPH radical-scavenging effects.

As shown in Figure 1, the free-radical scavenging activity of the BBEE was observed in the presence of the DPPH radical. It was found that the antioxidant activity of the BBEE is enhanced with the increase of the concentration. When the sample concentration was 100 *μ*g/mL, the inhibition rate exceeded 50%, reaching 52.92%. Therefore, the IC_50_ value of scavenging free radicals is 100 *μ*g/mL. Its inhibitory rate could reach 75% at the concentration of 2 mg/mL.

### 3.3. Tyrosinase Activity Inhibition

**Figure 2:**
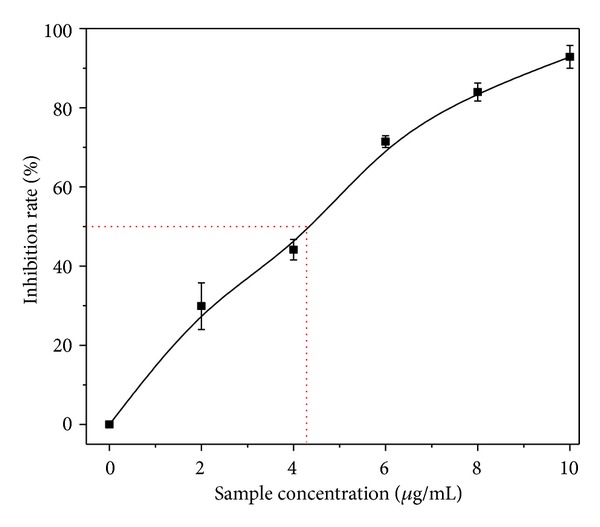
Tyrosinase inhibition of the sample.

In order to explain the inhibition of tyrosinase activity, we used the mushroom tyrosinase to test the activity inhibition of the BBEE. The results showed in Figure 2 that the tyrosinase inhibitory activity of the BBEE using L-tyrosine as substrate was examined. The result showed that the BBEE had tyrosinase inhibitory activity, but the inhibition was slightly low as compared to the DPPH free-radical scavenging activity. When the sample concentration was 4.0 mg/mL the inhibitory activity of tyrosinase was slightly lower than the IC_50_ value. However, the inhibitory activity did not improve obviously with the increase of the concentration. When increasing the BBEE concentration up to 8 mg/mL, the inhibitory rate reached 93%, but it did not become higher as the concentration increased. This phenomenon may be due to the interference of sample color in the high concentration.

### 3.4. Inhibitory Effects of the Extracts on *α*-Glucosidase and Sucrase

The mulberry root has been employed as a component of the diuretic or cough medicine in oriental medicine. The mulberry leaves are also used for the combination with other herbs and have positive impact on the diabetes mellitus [22]. In recent years, the mulberry leave tea is getting attention in various countries in Asia as it is claimed to be an antidiabetic drink. Furthermore, the freeze-dried powder of the silkworm larvae, especially the 3rd instar larvae reared with mulberry leaves was also found to have the function of hypoglycemia. So, the extract from the mulberry branch barks may also inhibit the hydrolysis of disaccharide such as maltose and sucrose. Therefore, we used rat small intestine acetone powder to test inhibitory activity of the BBEE for both of the *α*-glucosidase and the sucrase, and the results were shown in Tables 2 and 3. As expected, the BBEE not only had much strong inhibition for *α*-glucosidase, but also had much high inhibition for sucrase. It can be found from Table 2 that the extract showed the significant effect on *α*-glucosidase enzymatic reaction in which the maltose was used as a substrate. When the concentration of the BBEE was 2 *μ*g/mL, the inhibition rate of the enzymatic reaction could reach 30%, and as the concentration was elevated one time, the inhibition could reach above 40%; when the concentration was increased to 20 *μ*g/mL the enzymatic reaction was inhibited mostly by the BBEE, and its inhibition rate increased by over 70%. The IC_50_ value of the BBEE could be calculated to be about 8 *μ*g/mL.

The inhibitory activity of the extract for sucrase was shown in Table 3. We could see that its inhibition of sucrase activity was much stronger than that of above enzyme. Adding 0.025 *μ*g/mL of the BBEE to the reaction system, the inhibition for sucrase could reach 10%. Its inhibitory activity is rapidly enhanced with its concentration; when the concentration increased by 10 times (0.25 *μ*g/mL), the inhibitory rate reaches over 50%. Therefore, the IC_50_ value of the sucrase inhibitory activity was about 0.24 *μ*g/mL which is much lower than that of *α*-glucosidase.

### 3.5. Kinetic Studies

**Figure 3:**
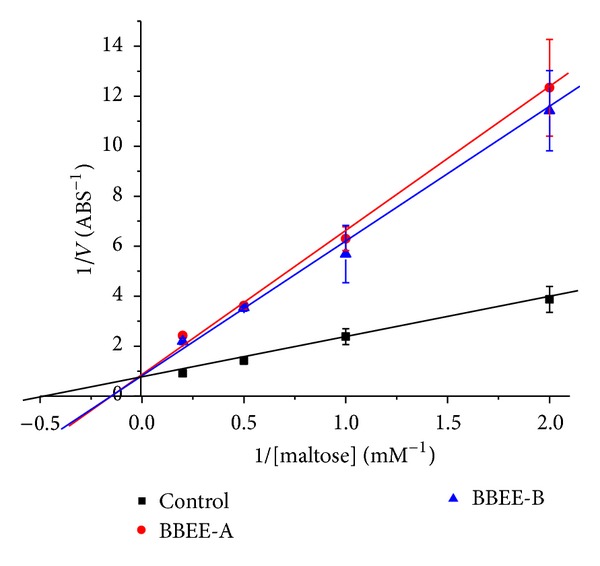
Lineweaver-Burk plots for kinetic analysis of *α*-glucosidase inhibition by the sample.

It has been reported that not only flavonoids but also iminosugar alkaloids DNJ [23] and polysaccharide existing in mulberry leaves had the inhibitory activity of the *α*-glucosidase. So these substances may also exist in the extract from the barks of mulberry branches. Therefore, in order to clarify the maltose inhibition mode of the extract to *α*-glucosidase, the test of the Lineweaver-Burk plots was performed (Figure 3). The black straight line means the kinetic curve of control group in which the *α*-glucosidase hydrolyzed in different concentrations of substrate maltose in reactive system without the BBEE. When the BBEE was added into the reactive system at two different concentrations of 1.95 and 1.56 *μ*g/mL (BBEE-A and BBEE-B in Figure 3), the hydrolysis of the *α*-glucosidase to maltose was obviously inhibited. It was found that the two lines (the blue and red lines in Figure 3) intersected at *y*-axis (0.92). The inhibitory activities of the extract of the mulberry branch barks were competitive against maltase as in the cases of the Acarbose [24] and DNJ [23]. It indicated that the BBEE would be a group of mixed *α*-glucosidase inhibitors containing not only N-containing sugars but also flavonoids and the other unknown inhibitors.

### 3.6. The Carbohydrate Tolerance

**Figure 4:**
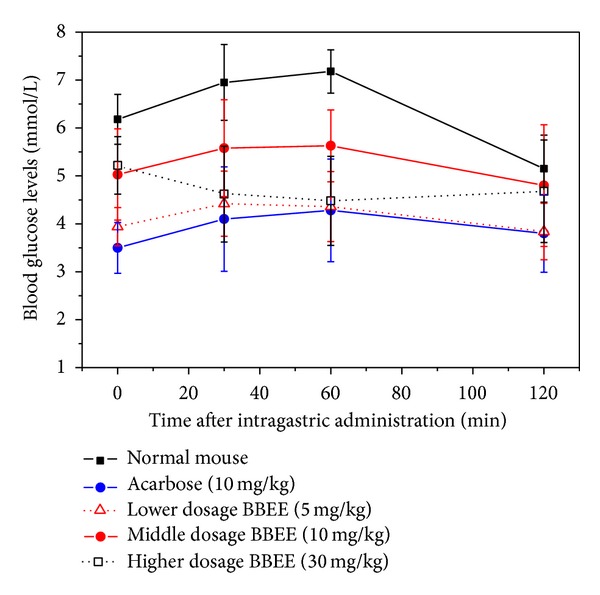
The carbohydrate tolerance of the normal mice with or without the BBEE.

The carbohydrate tolerance of the normal mice with or without the extracts was shown in Figure 4. The results clearly showed that the blood glucose levels (BGL) of negative control group continued to rise within 60 min after intragastric administration. The BGL began to decline after one hour and it is maintained at normal level after two hours due to the function of insulin. The BGL slowly rose in positive control group after being treated by the Acarbose with a dose of 10 mg/kg (BGL 30 min, 4.1 mM; BGL 60 min, 4.28 mM). These results clearly showed that the catalytic hydrolysis of *α*-glucosidase could be inhibited effectively by the Acarbose, and the speed of the hydrolysis from starch to glucose slowed down. The BBEE at 5, 10, and 30 mg/kg could significantly (*P* < 0.05) decrease the BGL as compared to the negative control group. The inhibition pattern of the BBEE at 5 and 10 mg/kg was the same as that of the Acarbose. The hydrolysis of *α*-glucosidase was inhibited excessively by the BBEE at 30 mg/kg. The BLG is maintained at a low level within 60 min (30 min 4.62 mM; 60 min 4.48 mM), and it returned at 2 h. Therefore, the hydrolysis of *α*-glucosidase could be inhibited effectively by the BBEE at 10 mg/kg. The glucose tolerance in normal mice could be improved by the BBEE.

### 3.7. Animal Experiments

**Figure 5:**
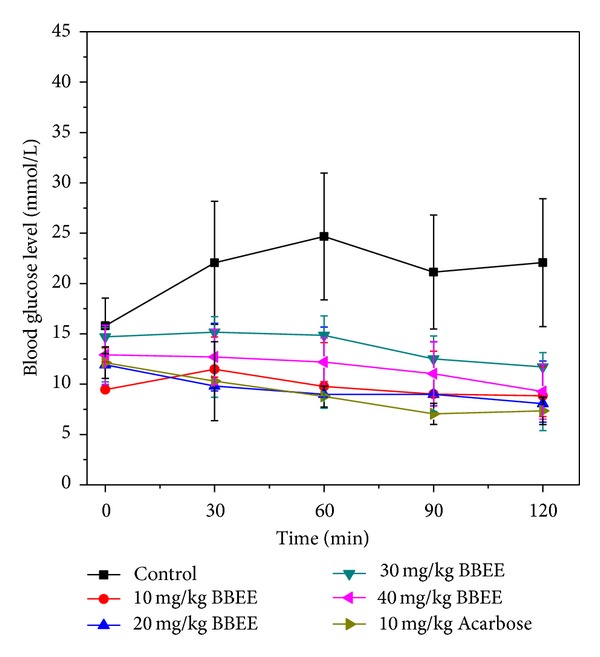
Effect of the BBEE oral administration (ig) on blood glucose level in starch administration.

A single oral administration test of the mulberry bark extracts with the potent *α*-glucosidase inhibitory activity was performed to clarify the antihyperglycemic effect *in vivo*. The animal experiments were done after administration of 10, 20, 30, and 40 mg/kg of the extracts, followed by at a dose of 10 g/kg starch in STZ-diabetic Kunming mice (the diabetic mice were induced with STZ, and the mice with fasting blood glucose level of above 7.8 mM were used for the experiment).  Figure 5 showed the change in BGL during the 120 min protocol in starch-loaded mice. The results clearly showed that the BGL of control group continued to increase within 60 min (BGL 30 min, 22.06 mM; BGL 60 min, 24.67 mM), and it declined slightly after 90 min (BGL 90 min, 21.14 mM; BGL 120 min, 20.07 mM). However, the BGL remained over 20 mM at 120 min. The BGL did not increase within 120 min after loading starch with the BBEE. It was shown that the hydrolysis of the *α*-glucosidase continued to be inhibited significantly (*P* < 0.01) *in vivo* by the BBEE.

## 4. Discussion

At present, the “mulberry twig particle”, a single herb extract from mulberry branches with a hypoglycemic effect, has been approved by the FDA of China and also has been clinically used [25]. The efficacy of each packet of the granules (3 g) is equivalent to one tablet of Acarbose containing the active ingredient 50 mg; its advantage has no the gastrointestinal side effects like Acarbose. In this experiment with the STZ-diabetic mice, the results show that a dose of 20 mg/kg of the BBEE on postprandial hypoglycemic effect is similar to a dose of 10 mg/kg of Acarbose and 600 mg/kg of the “mulberry twig particle” (data not shown) [26]. In other words, the hypoglycemic effect of the mulberry branch bark extract in this experiment is 30 times higher than the mulberry twig particles. The medical development on the alcohol extracts from mulberry branch bark has a simple preparation, rich source, and high yield. The low cost of the preparation will have an advantage more than the “mulberry stalk particles” or Acarbose. Therefore, the alcoholic extract of mulberry branch bark as a novel *α*-glucosidase inhibitor used in the treatment of diabetes has the potential development value in those countries where a large scale of cultivated mulberry has been planted.

The major components of the mulberry branch bark ethanol extract were the flavonoids, phenols, saccharides, and flavonoids glycosides. The 1-deoxynojirimycin (DNJ) is the type of the aza-sugar which has been found in the bark of the mulberry branch [27]. And research showed that the DNJ was a strong *α*-glucosidase inhibitor [28]. But the study suggested that there was no obvious relationship between *α*-glucosidase inhibitory activity and the 1-deoxynojirimycin content [29]. The ethanol extract as a competitive inhibitor could strongly inhibit the *α*-glucosidase activity whose IC_50_ value was 8.0 *μ*g/mL for maltose as a substrate, and it also could strongly inhibit sucrase whose IC_50_ value was 0.24 *μ*g/mL for sucrose. It also had a good DPPH radical scavenging activity with around 100 *μ*g/mL of IC_50_ value and a slight inhibition activity of the tyrosinase. Furthermore, the carbohydrate tolerance of the normal mice was obviously (*P* < 0.05) enhanced after the intragastric administration of the BBEE. And the postprandial hyperglycemia of the type 2 diabetic mice can be significantly decreased (*P* < 0.01) by the intragastric supplying of various concentrations of the BBEE (10~40 mg/kg).

## Figures and Tables

**Table 1:** Total flavonoids, phenolic and saccharides contents in the extracts.

Active components	Contents (*μ*g/g)
Total flavonoids	262.16 ± 8.57
Total phenols	118.70 ± 2.11
Total saccharides	75.88 ± 2.58

**Table 2:** *α*-Glucosidase inhibitory activities (maltose) of the samples with different concentration.

Sample con. (*μ*g/mL)	Inhibition rate (%)	±SD
1.67	30.47	2.82
2.50	31.88	2.08
4.00	43.35	1.10
5.00	43.73	0.74
20.00	73.52	3.93
50.00	81.37	2.43

**Table 3:** Sucrase inhibitory activities (sucrose) of the sample with different concentration.

Sample con. (*μ*g/mL)	Inhibition rate (%)	±SD
0.025	8.46	6.48
0.050	12.56	6.42
0.100	33.31	4.13
0.250	49.97	1.27
5.000	65.63	1.43
10.000	77.46	1.55
